# Undigested Pills in Stool Mimicking Parasitic Infection

**DOI:** 10.1155/2017/3718954

**Published:** 2017-01-31

**Authors:** Fazia Mir, Ilyas Achakzai, Jamal A. Ibdah, Veysel Tahan

**Affiliations:** ^1^Division of Gastroenterology and Hepatology, University of Missouri, Columbia, 1 Hospital Drive, Columbia, MO 65212, USA; ^2^Department of Internal Medicine, Erie County Medical Center, 1 John James Audubon Pkwy, Buffalo, NY 14228, USA

## Abstract

*Background*. Orally ingested medications now come in both immediate release and controlled release preparations. Controlled release preparations were developed by pharmaceutical companies to improve compliance and decrease frequency of pill ingestion.* Case Report*. A 67-year-old obese male patient presented to our clinic with focal abdominal pain that had been present 3 inches below umbilicus for the last three years. This pain was not associated with any trauma or recent heavy lifting. Upon presentation, the patient reported that for the last two months he started to notice pearly oval structures in his stool accompanying his chronic abdominal pain. This had coincided with initiation of his nifedipine pills for his hypertension. He reported seeing these undigested pills daily in his stool.* Conclusion*. The undigested pills may pose a cause of concern for both patients and physicians alike, as demonstrated in this case report, because they can mimic a parasitic infection. This can result in unnecessary extensive work-up. It is important to review the medication list for extended release formulations and note that the outer shell can be excreted whole in the stool.

## 1. Background

Orally ingested medications have undergone a transformation over the years. In order to improve compliance by decreasing frequency, the pharmaceutical industry developed controlled release formulations. Immediate release medications usually need to be dosed frequently to maintain blood levels or usually can cause gastrointestinal irritation. As controlled release pill formulations are released slowly, the outer capsule shell may be seen undigested in the stool. This may be a source of considerable anxiety for both the physician and the patient. Herein we present a case of a male patient who was passing “pearly oval structures” that resembled a parasitic ovum. This caused a lot of alarm for the patient which resulted in him receiving unnecessary investigations.

## 2. Case Report

**Figure 1:**
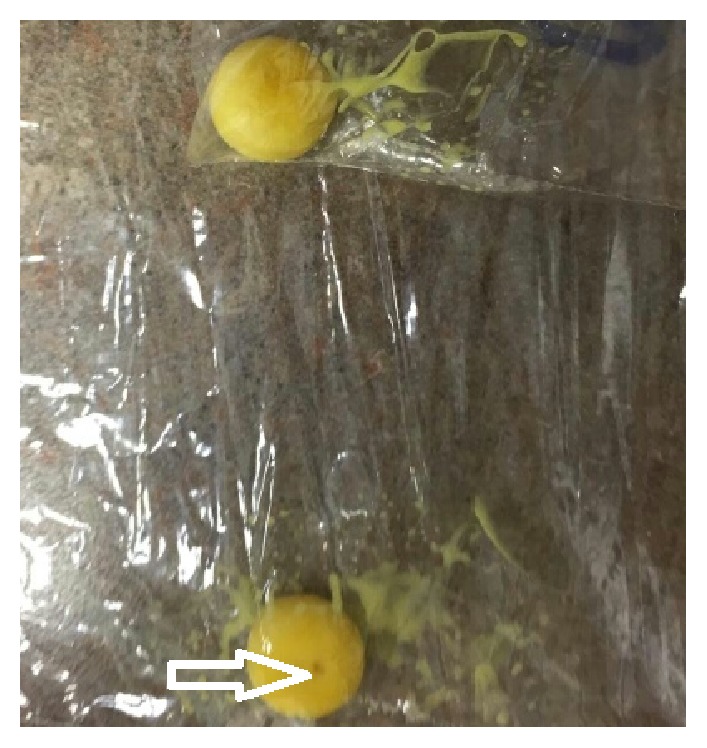
Pearly structures in the stool. The arrow shows a central hole.

**Figure 2:**
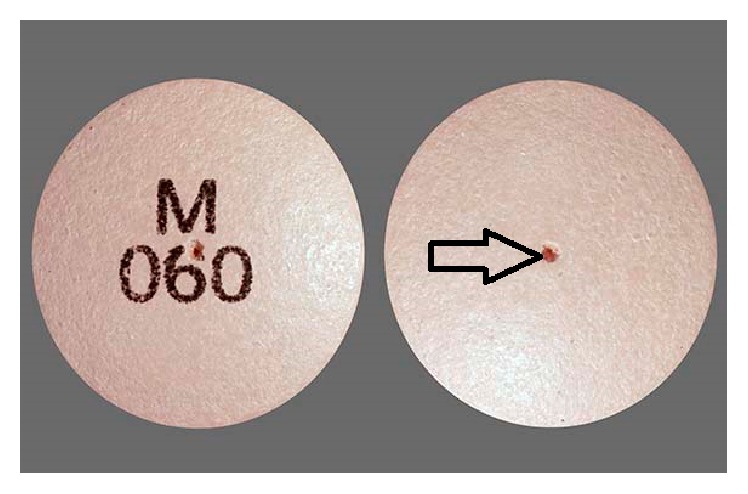
An original nifedipine pill with a hole for sustained release effect.

A 67-year-old obese male patient presented to our clinic with focal abdominal pain that had been present 3 inches below umbilicus for the last three years. This pain was not associated with any trauma or recent heavy lifting. Upon presentation, the patient reported that for the last two months he started to notice pearly oval structures in his stool accompanying his chronic abdominal pain. The patient reported no history of recent travel inside or outside the country, no well water ingestion, or any camping trips. He denied smoking, alcohol, or substance abuse. He reported no family history of inflammatory bowel disease or colon cancer. He denied that he had ingested any foreign body. His laboratory work-up including a complete blood count, complete metabolic panel, and a computed tomography scan was unrevealing for pathology. He continued to report passage of these “pearly oval structures” in his stools on a daily basis. Our differential included ingestion and passage of foreign body, parasitic infection, or undigested pills. Stool studies were conducted on three separate visits looking for ova and parasites but were negative. He previously had a colonoscopy by a community gastroenterologist which was noted to be normal with no sight of these “pearly oval structures.” The patient was then asked to bring these “pearly structures” to the clinic upon his next visit (Figure 1). The medication history was revisited, but this time it was done in liaison with a pharmacist. The pharmacist noted that the “pearly oval structures” were his nifedipine extended release pills (Figure 2) that were passing undigested through his alimentary tract.

## 3. Discussion

With the advent of extended release formulations, there have been times where these extended release capsules are excreted in stool. There has been a case of fecal impaction with ingestion of enteric coated pills [1]. A study of Taiwanese HIV patients excreting remnants of nevirapine extended release tablets did not report any decrease in efficacy of the medicine despite the presence of parts of undigested tablets in patients' stool [2].

Nifedipine is a calcium channel blocker given for treatment of hypertension and chronic angina, with frequent dosing in the past [3]. An extended release formulation of nifedipine was developed to achieve once-daily dosing using the gastrointestinal therapeutic system (GITS) [4]. Nifedipine using GITS has an internal component and external component. The internal component contains the drug surrounded by a hydrophilic polymer with a single hole that is semipermeable. This semipermeable hole enables water to enter the drug layer, causing the pill to swell and pushing the nifedipine powder out a little at a time. This system controls the release of nifedipine at a constant rate over 24 hours until there is no powder left to be extruded. The outer membrane of the tablet, which is made of a hydrophilic polymer, is resistant to digestion, remains intact throughout the alimentary tract, and is excreted in the stool [3].

## 4. Conclusions

The undigested pills may pose a cause of concern for both patients and physicians alike, as demonstrated in this case report, because they can mimic a parasitic infection. This can result in unnecessary extensive work-up. It is important to review the medication list for extended release formulations and note that the outer shell can be excreted whole in the stool.
